# Hitching a Ride: Examining the Ability of a Specialist Baculovirus to Translocate through Its Insect Host’s Food Plant

**DOI:** 10.3390/pathogens10111500

**Published:** 2021-11-18

**Authors:** Peter P. Issa, Michael Garvey, Scott Grimmell, Pramod Pantha, Maheshi Dassanayake, Bret D. Elderd

**Affiliations:** Department of Biological Sciences, Louisiana State University, Baton Rouge, LA 70803, USA; pissa1@lsu.edu (P.P.I.); sgrimm5@lsu.edu (S.G.); ppanth1@lsu.edu (P.P.); maheshid@lsu.edu (M.D.); elderd@lsu.edu (B.D.E.)

**Keywords:** *Spodoptera frugiperda*, SfMNPV, baculovirus, nuclear polyhedrosis virus, *Zea mays*

## Abstract

Plant vascular systems can translocate the entomopathogen *Bacillus thuringiensis* from the soil into plant tissues. However, whether other soil dwelling entomopathogens utilize plant vascular tissue for movement has not yet been fully explored. We used *Spodoptera frugiperda* multiple nucleopolyhedrovirus (SfMNPV) to evaluate whether baculoviruses, a common entomopathogen and bioinsecticide, can be transported through the plant vascular pathways of *Zea mays*. We found that our treatments did not allow a sufficient virus translocation into the plant to induce a lethal infection in insects, which was confirmed by a molecular analysis. While other entomopathogens translocate, baculoviruses may not be one of them.

## 1. Introduction

Plant pathogens are not alone in their ability to enter and navigate through a plant host. The common commensal soil bacterium *Bacillus thuringiensis* has been shown to possess the ability to be admitted and translocated by the plant vascular tissue via the phloem [1,2]. Entomopathogenic fungi, such as *Beauveria spp*. and *Metarhizium spp*., are also known to have endophytic associations with plants [3]. Uptake into plants allows these microscopic species to escape ultraviolet light exposure, which degrades their viability [4,5], allowing the microbe to persist in the environment for longer. More importantly though, translocation from the rhizosphere to foliar tissue allows the pathogen to infect potential insect hosts [1,2,3]. Thus, this microbe-plant-insect interaction has the potential to be a co-evolutionary partnership which would have obvious applications in agriculture. The breadth and complexity of these plant-entomopathogen interactions across other entomopathogen groups, such as viruses is however unclear. To explore this further, we chose to examine if plant vasculature systems could admit and translocate entomopathogenic viruses using *Zea mays* (maize), *Spodoptera frugiperda* (the fall armyworm), and the specialist nuclear polyhedrosis virus (NPV) *Spodoptera frugiperda* multiple nucleopolyhedrovirus (SfMNPV).

*Spodoptera frugiperda* is a generalist insect pest with a preference for grains, causing devastating widespread economic and ecological damage globally [6,7]. SfMNPV is a specialist baculovirus of *S. frugiperda*, which resides in the soil and on contaminated plant foliage in the form of occlusion bodies (OBs). The horizontal transmission of SfMNPV depends on rain, wind, or a host for dispersal. Acquisition per os of a lethal dose by the larval phase of the herbivore leads to an infection, with infected individuals ceasing to grow, swelling from a budded virus and then forming OBs, before lysing, which reintroduces the virus back into the environment [8].

We examined the possibility of SfMNPV admittance and translocation within maize in the hope of uncovering a novel route of infection of this virus to its insect host. We experimentally examined SfMNPV admittance into maize via two routes to mimic natural conditions: entry by means of a natural opening (roots) and entry by means of a shear cut (cut leaves). For the former, we drenched the soil of maize plants with a suspension of the virus in water, and for the latter, we placed leaf cuttings in the same viral water suspension. In addition, this combination of treatments allowed us to determine whether the virus could be translocated via the roots to the leaf tissue or if the virus was able to be translocated via the petiole of the leaf.

## 2. Results

**Figure 1:**
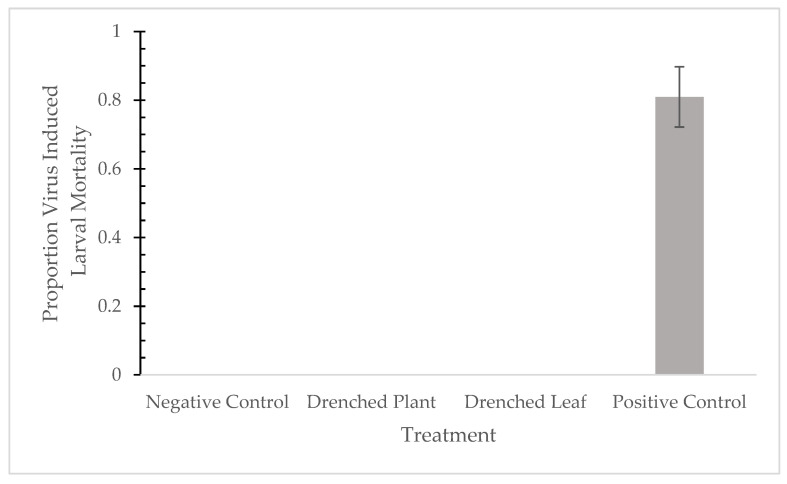
Application method of baculovirus on fall armyworm virus-induced mortality (± standard error). Bars show the percent of fall armyworms that were infected when the baculovirus application varied. All virus water suspensions had the same concentration.

The percentage of infected fall armyworms was unaffected when neonates were fed plants or leaves drenched in the virus water suspension (Table 1). In contrast, the positive control was able to induce an 86% infection rate (Table 1 and Figure 1), whereas no infections occurred in the negative controls.

**Figure 2:**
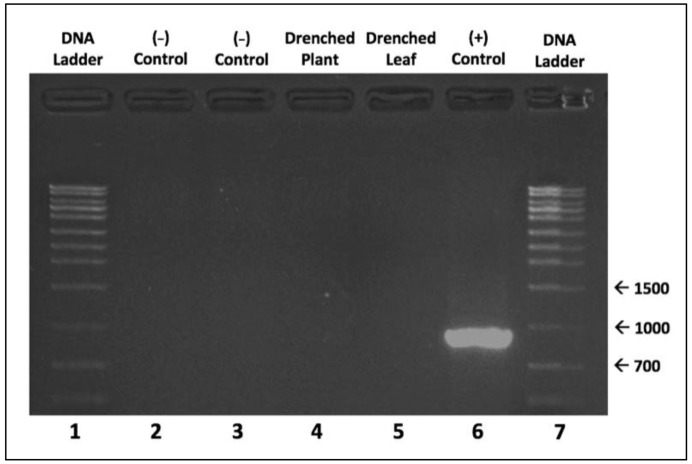
Gel electrophoresis run on samples from each treatment. Lane 1: 1 kb DNA ladder; Lane 2: Negative Control; Lane 3: Negative Control; Lane 4: Drenched Plant; Lane 5: Drenched Leaf; Lane 6: Positive Control; Lane 7: 1 kb DNA Ladder.

Furthermore, our molecular analysis confirmed the presence/absence of viral DNA according to the baculovirus application method. The presence of viral DNA was detected solely in the positive control treatment, while viral DNA was not detected in the negative control, drenched plant, or drenched leaf treatments (Figure 2). Taken together, our results do not support the possibility of the uptake and translocation of SfMNPV within the plant.

## 3. Discussion

Overall, we were unable to infect larvae with the baculovirus via translocation nor amplify viral DNA from the manipulated treatment groups (Figure 1 and Figure 2). The results of the bioassay were consistent with the data from the molecular analysis and suggested that the virus water suspension was neither taken up nor translocated through the plant vascular system. Root uptake and translocation within the plant can occur primarily by two pathways, (i) short-distance intercellular transport and (ii) long-distance transport using the xylem/phloem. These pathways involve cellular transport within plant cells governed by plasmodesmata or vesicular trafficking and involve channel proteins that facilitate macromolecule transport [9]. Viral-encoded movement-proteins are occasionally known to interact with plant channel proteins to allow virus movement from cell to cell [10,11]. Although baculoviruses are capable of infecting animal cells [12,13], they are not known to be pathogenic to plants [8]. Therefore, a direct opportunity for selection to allow non-pathogenic viral transport within plants is absent. Our current results imply that maize plants are unable to uptake SfMNPV, but there is still more work to be done. Further examination of these interactions across plant species, insect herbivores, and different types of entomopathogens will be vital to determine the complexity and breadth of these relationships in nature. In particular, granuloviruses could be particularly interesting since they are small, nonoccluded viruses, making them perhaps capable of translocation in different plant tissues. As for now, though, while translocation is a possibility for *B. thuringiensis*, the window is closed for the translocation of SfMNPV in maize.

## 4. Materials and Methods

*Material and Experimental setup*: To test baculovirus admittance into and through the plant vascular system, *Zea mays* cv. Early Sunglow (NE Seed; East Hartford, CT, USA) plants were grown from seeds under laboratory conditions in bioclimatic chambers (Conviron; Winnipeg, MB, Canada) at 80% humidity, 28 °C, and on a 16:8 h photoperiod individually in 15.24 cm pots. Seeds were planted in soil containing a 2:1:1 ratio of A1 Soil Mix (SunGro Horticulture, Agawam, MA, USA) and top-dressed with 5 mL Osmocote 14-14-14 slow release fertilizer (The Scotts Miracle-Gro Company, Marysville, OH, USA). Plants were grown for a total of 15 days before being used in the subsequent experiment.

We used a commercially available SfMNPV, Fawligen (strain number MNPV-3AP2, lot number S190405; AgBiTech; Fort Worth, TX, USA), for all experiments; ~33% of the product contains OBs, with the rest being made up of a water/glycerol mixture. The commercial preparation was diluted to 1.5 × 10^4^ OBs/μL—the label recommendation for the concentration in foliar field applications on *Z. mays*. We randomly selected maize plants, covered their base in plastic wrap to prevent splashing, and watered them from the top of the soil surface with 200 mL of the viral water suspension daily for four days before the start of the subsequent mortality bioassay. This amount is the equivalent of ~100 infected 4th instar larvae per day. These plants comprised our drenched soil whole plant treatment. Our second treatment consisted of the cut leaf group to test the ability of the baculovirus to enter via the petiole. We randomly selected plants, cut 2.5 cm above their base in the soil, and placed the leaves in a 10 cm floral water pick/tube (Oasis Floral Products, Kent, OH, USA) that contained 15 mL of the virus water suspension. These leaves were placed in a 28 °C incubator, and their virus water suspension was replaced daily for two days before use in the subsequent mortality bioassay.

*Neonate Mortality Bioassay*: Viral translocation was tested using a mortality bioassay. Plant foliage (enough whole plant leaves for larvae to feed ad libitum) from each treatment (n = 12 per treatment; controlled for plant size) was placed in closed 100 × 15 mm petri dishes containing filter paper discs, saturated with water daily to prevent the leaf tissue from drying out, during the experiment. As a positive control, foliage was taken from the control plants and dipped into a 1.5 × 10^4^ OBs/μL virus water suspension (n = 5). For the negative control, control plant foliage was left untreated and also placed into petri dishes containing water-saturated paper discs. For all treatments, five newly hatched neonates (Benzon Research Inc., Carlisle, PA, USA) were then placed into each petri dish and incubated at 28 °C to be reared on plant material for four days. Larvae that are lethally infected arrest development and stop feeding after 24 h before dying ~3 days later given this life stage/viral dose combination [13]. To determine if larvae died of a baculovirus infection, dead larvae were autopsied and examined under a bright field microscope at a 20× magnification. Confirmation of OBs was determined if OBs lysed upon the addition of a 1 M KOH solution [14].

*Molecular Confirmation of Virus Translocation within Plants*: Plant samples (whole leaves) from each of the aforementioned treatment groups (24 per treatment, 2 for each of the 12 plant individuals in a treatment) were collected and subsequently flash frozen in liquid nitrogen and stored at −82 °C. The day of DNA extraction, tissue samples were vortexed for ten minutes with Lysing Matrix A beads (MP Biomedicals; Santa Ana, CA, USA) and ground with a micropestle for two minutes to achieve homogenization. DNA was extracted from 80 mg of leaf tissue using the DNeasy Plant Mini Kit, following manufacture guidelines (Qiagen; Hilden, Germany). Gene specific forward (5ʹ CAAGCCGGAACTCGTGTATAG 3ʹ) and reverse primers (5’ ATGACCGTTTGAGGCAGATAG 3’) were designed for SfMNPV gene sf72 using the PrimerQuest Tool (https://www.idtdna.com/Primerquest accessed on 10 February 2020) based on the SfMNPV genome [15] and purchased from Integrated DNA Technologies (Coralville, IA, USA). SfMNPV gene sf72 encodes for ABM45782.1, a DNA helicase with the total length amplified being 996 nucleotides.

Extracted DNA was diluted to a concentration of 12.5 ng/μL in a PCR master mix (i-MAX II; iNtRON Biotechnology, South Korea) with gene specific primers in a total volume of 20 μL. PCR amplification was conducted using a BioRad T100 Thermal Cycler (Hercules, CA, USA) with an initial denaturation set at 94 °C for 3 min, followed by 30 cycles of denaturation at 94 °C for 30 s, annealing at 60.8 °C for 30 s, extension at 72 °C for 3 min, and ending in a final extension at 72 °C for 10 min. The PCR assay was optimized beforehand using a gradient heating technique to optimize the annealing stage to find the optimal temperature for the amplification product yield.

PCR products were run on a VWR Mini Gel II Electrophoresis System (Radnor, PA, USA). 5 μL of amplified product was run at 80 volts for 50 min in a 1.5% agarose gel (Agarose Unlimited; Gainesville, Fl, USA) with Tris-acetate buffer (TAE). Amplified products were stained with RedSafe (iNtRON Biotechnology; Seongnam-si, Gyeonggi-do, South Korea) and imaged using a Bio-Rad Molecular Imager (Hercules, CA, USA).

*Statistical Analysis*: Statistical analysis was conducted using the R statistical software 4.0.2 [16]. Insect mortality due to plant treatment was analyzed using a generalized linear mixed effect model (GLMM) with the group insect enclosure (i.e., the petri dish) used as a random effect and the virus application as the fixed effect in the lme4 package [17]. The GLMM was fit with a binomial distribution, with the positive control (virus application on the surface of the plant) used to weight the model.

## Figures and Tables

**Table 1:** GLMM parameter estimates for the effect of each treatment on the probability of *S. frugiperda* virus-induced mortality. The intercept corresponds to the positive control where the foliage was dipped in a virus water suspension. The three other terms correspond to when larvae were fed on untreated plants (i.e., negative control), plant soil was drenched in a virus water suspension (i.e., drenched plant) and leaves were drenched in a virus water suspension (i.e., drenched leaf). All virus water suspensions had the same concentration.

Term	Estimate	Standard Error	Z Value	Pr (>|z|)
Intercept	1.447	0.556	2.604	0.009
Negative Control	−39.320	1.036 × 10^7^	0.000	1.000
Drenched Plant	−38.690	8.523 × 10^6^	0.000	1.000
Drenched Leaf	−46.370	9.789 × 10^6^	0.000	1.000

## Data Availability

Data for the associated described experiments are available from the corresponding author upon request.
